# Outcome of Elderly Patients with Venous Thromboembolism Treated with Direct Oral Anticoagulants—A Retrospective Cohort Study

**DOI:** 10.3390/jcm10235673

**Published:** 2021-12-01

**Authors:** Irit Ayalon-Dangur, Yakov Vega, Miriam Rozi Israel, Alon Grossman, Galia Spectre, Tzippy Shochat, Leonard Leibovici, Anat Gafter-Gvili

**Affiliations:** 1 Internal Medicine E, Rabin Medical Center, Beilinson Campus, Petah Tikva 49100, Israel; iritayalon1234@gmail.com (I.A.-D.); Miroz187@gmail.com (M.R.I.); Leibovici@clalit.org.il (L.L.); 2 Sackler Faculty of Medicine, Tel Aviv University, Tel Aviv 69978, Israel; yakovega@gmail.com (Y.V.); ALONG@clalit.org.il (A.G.); galiasp1@clalit.org.il (G.S.); 3 Internal Medicine B, Rabin Medical Center, Beilinson Campus, Petah Tikva 49100, Israel; 4 Institute of Hematology, Davidoff Cancer Center, Rabin Medical Center, Beilinson Campus, Petah Tikva 49100, Israel; 5 Bio-Statistical Unit, Rabin Medical Center, Beilinson Campus, Petah Tikva 49100, Israel; tzippysh@clalit.org.il; 6 Internal Medicine A, Rabin Medical Center, Beilinson Campus, Petah Tikva 49100, Israel

**Keywords:** elderly, venous thromboembolism, deep vein thrombosis, pulmonary embolism, direct oral anticoagulants, vitamin K antagonists, enoxaparin

## Abstract

Introduction: Randomized controlled trials that compared direct oral anticoagulants (DOACs) to vitamin K antagonists (VKA) for the treatment of venous thromboembolism (VTE), demonstrated both efficacy and safety of DOACs. The aim of the current study was to compare DOACs to VKA for the treatment of VTE in the elderly, in a real-life setting. Methods: A retrospective cohort study was performed in Rabin Medical Center encompassing a 7-year period. Hospitalized patients >65 years, with a diagnosis of VTE discharged with DOACs or VKA were included. The primary outcome was a composite of all-cause mortality, major bleeding, recurrent VTEs and hospitalizations throughout the follow-up period of one year. Results: A total of 603 patients were included in the final analysis. The mean age was 79.6 ± 8.5 years. The primary composite outcome occurred in 74.6% and 56.7% of the patients in the VKA group and DOACs group, respectively, hazard ratio 0.59, 95% confidence interval 0.46 to 0.76, in favor of the DOACs group. In a matched cohort analysis, the results were the same as the original analysis. Conclusion: In the elderly population, treatment of VTE with DOACs was associated with a lower rate of the composite outcome. DOACs are safe and effective for elderly patients with VTE.

## 1. Introduction

Venous thromboembolism (VTE) is the third most common cause of morbidity and mortality from cardiovascular disease after myocardial infarction (MI) and stroke. The annual incidence of VTE is estimated to be around 1.5–3.0/1000 cases [1,2]. The conventional therapy until recently was parenteral treatment with low molecular weight heparin (LMWH) for at least five days and vitamin K antagonists (VKA) as warfarin during this time period and continued for a minimum of three months [3]. Large randomized controlled trials that compared direct oral anticoagulants (DOACs) to VKA for the treatment of VTE—including AMPLIFY, which assessed apixaban; RE-MEDY, which assessed dabigatran; EINSTEIN, which assessed rivaroxaban; and Hokusa-VTE, which assessed edoxaban—demonstrated both efficacy (non-inferiority regarding the primary efficacy outcome of recurrent VTE or VTE related death) and safety of DOACs [1,4,5,6,7,8]. Based on these trials, the American College of Chest Physicians (ACCP, CHEST) 2016 guidelines recommend the use of any of the DOACs over VKA treatment (grade 2B recommendation) [9]. The European Society of Cardiology (ESC) guidelines for the diagnosis and management of acute pulmonary embolism recommend that when oral anticoagulation is initiated in a patient with PE who is eligible for a DOACs, a DOACs is the recommended form of anticoagulant (grade 1 recommendation) [10]. The American Society of Hematology (ASH) 2020 guidelines for management of venous thromboembolism suggest using direct oral anticoagulants over vitamin K antagonists as well [11].

VTEs are more prevalent in the elderly. The risk for VTE is four to six times greater in patients above the age of 70 years than the risk in patients younger than 70 [12], and the risk for VTE is doubled with each decade [13]. In addition to the higher prevalence of VTEs in the elderly, the bleeding risk is increased as well. The risk of major bleeding in patients using anticoagulants is 2.5% per year in patients older than 80 years compared to 0.9% per year in patients younger than 80 [14,15].

In the three large trials quoted above [1,4,5] the mean age examined was 55 years (SD 15). Patients older than 70 years were included, yet the percentage of elderly patients over the age of 75 was negligible (in the AMPLIFY, RE-COVER and EINSTEIN trials, 14%, 10.4% and 18%, respectively, representing about 2500 patients in total). A systematic review and meta-analysis which extracted data from the AMPLIFY, RE-MEDY, EINSTEIN and Hokusai trials found that DOACs showed the same or greater efficacy compared with vitamin K antagonists in elderly patients older than 75 years old [16]. Another large systematic review, based on the same studies found that treatment with DOACs is as effective as warfarin and a safer alternative in an elderly population [17].

Nevertheless, no RCTs were conducted specifically in the elderly population. Patients included in RCTs do not necessarily represent patients seen in daily clinical practice.

The aim of the current study is to compare DOACs to LMWH/VKA in terms of efficacy and safety, for the treatment of VTE in the elderly, in the real-life setting.

## 2. Materials and Methods

### 2.1. Study Design

A retrospective cohort study was performed in Rabin Medical Center encompassing a 7-year period from January 2012 to December 2018. The study was approved by the Rabin Medical Center (RMC) institutional review board (confirmation number 0165-18-RMC). The trial was conducted in accordance with the principles of the Declaration of Helsinki. Our study is retrospective and therefore informed consent was waived by the institutional review board.

### 2.2. Study Population

All patients > 65 years old, who were hospitalized with either a symptomatic, confirmed diagnosis of new lower extremity deep vein thrombosis (DVT) and/or pulmonary emboli (PE) and who were either discharged with DOACs (rivaroxaban, apixaban or dabigatran) or LMWH/VKA were included in the analysis. Patients who developed DVT or PE during the hospitalization were excluded. Patients with a contraindication to anticoagulation (i.e., active bleeding, platelet count < 50,000/microL, major trauma, history of intracranial hemorrhage (ICH)) were excluded. The diagnosis of either DVT or PE was confirmed by imaging studies of lower extremity (Doppler ultrasound for DVT) and CT angiography or perfusion scan for PE. The final cohort was composed of two groups of VTE patients depending on the medical therapy that the patient received: LMWH/VKA vs. DOACs, including apixaban, rivaroxaban and dabigatran.

### 2.3. Data Collection, Surveillance and Follow-Up

Data were collected from the computerized system in RMC. Demographic variables, vital signs on admission, relevant laboratory values including D dimer, imaging study, final diagnosis of DVE and/or PE, a history of VTE, treatment in the ED and following the discharge with LMWH and VKA or DOACs, duration of hospitalization, co-morbidities according to the Charlson comorbidity index [18] and previous anti-aggregation and anticoagulation medications were extracted from the patients’ computerized medical records.

Patients were followed-up for one year following the hospital admission, for the occurrence of outcomes. Data regarding the type of anticoagulant used for treatment were retrieved from the patients’ files and the hospitalization discharge letter. We collected data regarding medications after discharge dispensed by the pharmacy.

### 2.4. Outcome Assessments

The primary outcome was a composite outcome of all-cause mortality, major bleeding events, recurrent VTEs and hospitalizations throughout the FU period of one year. Date of death was retrieved through the hospital administration system, which is updated with Israel’s Ministry of Interior data. Secondary outcomes included each of the following: recurrent VTEs, major bleeding events and all-cause mortality in one-year FU, as well as a composite of recurrent VTEs, major bleeding events and all-cause mortality. Major bleeding was defined according to the ISTH definitions as overt bleeding with a decrease in the hemoglobin level of a 2 or more g/dL, a bleeding that required blood transfusion of more than two units of blood, occurred into a critical site or contributed to death [19].

### 2.5. Statistical Analysis

The statistical analysis was generated using SAS Software, Version 9.4 (SAS Institute, Cary, NC, USA). Continuous variables were presented by mean ± standard deviation (std). T-Test was used to compare the value of continuous variables between study groups and Fisher’s exact test (for two groups) or Chi-square (for more than two groups) were used to compare the value of categorical variables between study groups. Overall survival was assessed by the Kaplan–Meier model, with the log-rank test. The Cox Proportional Hazards model was used to calculate hazard ratios (HR). A multivariable analysis was constructed according to univariable analysis by entering variables that were statistically significant in the univariable analysis, as well as age and gender. Propensity score matching was used to create a matched cohort for the primary outcome. The factors for the propensity analysis were chosen based on comorbidities, as malignancy, on parameters of clinical importance and parameters that may influence the risk for VTE. The model was used to match between the group receiving VKA/LMWH and those that received DOACs using a logistic regression model. The analysis was performed using a caliper equal to 0.2 and based on a 2:1 ratio.

For the secondary endpoints of recurrent VTEs and major bleeding, the Fine and Gray methodology was used to adjust the HR, for the competing risk of death. Medication was included in the Cox model as a time dependent covariate. Two-sided *p* values less than 0.05 were considered statistically significant.

## 3. Results

### 3.1. Study Population

Overall, 603 patients were identified and included in the final analysis. A total of 476 patients were treated with LMWH/VKA and 127 patients were treated with DOACs. For 42 patients the medication was switched: 39 patients were switched from VKA/LMWH to DOACs and three from DOACs to VKA/LMWH. Baseline characteristics of patients included in the final analysis are presented in Table 1.

The mean age was 79.9 ± 8.5 years in the VKA/LMWH group and 78.6 ± 8.1 years in the DOACs group, *p* = 0.11. The duration of hospitalization was longer in the LMWH/VKA by 1.4 days compared to the DOACs group (7.3 ± 7.2 vs. 5.9 ± 4.9, respectively, *p* = 0.01). The co-morbidities according to the Charlson comorbidity index were similarly distributed in the two study groups, except for known malignancy that was more prevalent in the VKA/LMWH group with 20.1% in compared to 9.5% in the DOACs group. Previous VTEs events were also similarly distributed in the two study groups.

### 3.2. Clinical Outcomes during a Follow-Up Period of One Year

The number and percentage of events in each outcome are represented in Table 2.

The primary endpoint of a composite outcome of all-cause mortality, major bleeding, recurrent VTE and hospitalizations throughout the FU period occurred in 355/476 patients (74.6%) in the VKA/LMWH group and in 72/ 127 (56.7%) patients in the DOACs group, hazard ratio (HR) of 0.59, 95% confidence interval (CI) 0.46 to 0.76, *p* < 0.0001, in favor of the DOACs group. HR for recurrent VTEs and major bleedings events was 0.58 (95% CI 0.26 to 1.28) and 1.15 (95% CI 0.50 to 2.65), respectively, for the DOACs group, however without significance (*p* = 0.18 and *p* = 0.75, respectively). There was a significant reduction in mortality in the DOACs group-HR 0.30, 95% CI 0.20 to 0.44, *p* < 0.0001. Details regarding causes of death are provided in the Supplementary Table S1. Overall, 54 patients died from malignancy, which comprises 17.1% of causes of death. In the VKA/LMWH and DOACs groups 49 and five patients (16% and 31% of the mortality events in those groups, respectively) died from cancer. Another common cause of death was sepsis. Of note, there was no information regarding the cause of death in almost 50% of cases of death. HR for the composite outcome of recurrent VTEs, major bleeding events and all-cause mortality was 0.38 in favor of the DOACs group, 95% CI (0.27–0.55), *p* < 0.0001.

In a multivariable analysis for the primary outcome, treatment with DOACs, as well as age, elevated creatinine levels and malignancy were all associated significantly with the composite outcome of all-cause mortality, major bleeding, recurrent VTE and hospitalizations throughout the FU period of one year, HR of 0.64 (CI of 0.49–0.83) for the treatment with DOACs (Table 3).

In addition to the analysis above, we also conducted the same analysis on a matched cohort according to gender, age, prior MI, CHF, diabetic mellitus, renal disease, malignancy and prior VTE, in order to ensure that the differences between the groups had minimal influence on our results. In the matched groups there were 214 patients in the VKA/LMWH group and 107 patients in the DOACs group. The analysis on the propensity matched cohort produced the same results as the original analysis, as well for the multivariable analysis, with HR of 0.63 for the DOACs group in the composite outcome, 95% CI 0.47 to 0.86, *p* = 0.0032.

## 4. Discussion

In this retrospective, single-center study of 603 elderly patients with VTE, we assessed the efficacy and safety of DOACs, compared with the standard treatment of LMWH followed by VKA. The main finding is a lower rate of the composite outcome of all-cause mortality, major bleeding, recurrent VTE and hospitalizations in patients treated with DOACs compared to LMWH/VKA, with an HR of 0.59 (95% CI 0.46 to 0.76). These results were also statistically significant in a matched cohort produced by a propensity score analysis. This finding is consistent with the findings of previous studies in younger populations [1,4,5]. Regarding our secondary outcome, the HR for recurrent VTEs and major bleedings events was not significant, however there was a significant reduction in mortality in the DOACs group with HR of 0.30, 95% CI 0.20 to 0.44, *p* < 0.0001. Importantly, this is the first time that a reduction in mortality is demonstrated in the group that was treated with DOACs, which emphasizes the fact that in older age there is a considerable advantage in treatment with DOACs.

Notably, in our analysis there were relatively many mortality events in both groups compared to previous large randomized controlled trials, including the AMPLIFY, RE-MEDY, EINSTEIN and Hokusai-VTE trials [1,4,5,6], probably due to the older age of this cohort. However, the mean age was similar in both of our groups and mortality rate was significantly lower in the DOACs group.

The co-morbidities according to the Charlson comorbidity index were similarly distributed between the two study groups, except for malignancies that were more prevalent in the VKA/LMWH group, a finding that is not surprising because LMWH was considered the treatment of choice for VTE in patients with known active malignancy at that time (according to the American College of Chest Physicians (ACCP, CHEST) 2016 guidelines LMWH over VKA therapy was grade 2B, DOACs was grade 2C) [9]. The safety and efficacy of DOACs in cancer-associated thrombosis was thoroughly studied only later, in several trials that demonstrated noninferiority of DOACs: the HOKUSAI trial that evaluated edoxaban [20], the SELECT D published in 2018, that evaluated rivaroxaban [21] and the CARAVAGGIO trial (March 2020) that evaluated apixaban [21]. Current guidelines now recommend specific DOACs (edoxaban or rivaroxaban) to be considered as an alternative to LMWH, with the exception of patients with gastrointestinal cancer [10]. These guidelines do not include the recommendation to consider the use of apixaban because the publication of the CARAVAGGIO study [22] was subsequent to the publication of the current guidelines.

In a multivariable analysis, treatment with DOACs (compared with LMWH/VKA) as well as age, elevated creatinine levels and malignancy were all associated significantly with the composite outcome of all-cause mortality, major bleeding, recurrent VTE and hospitalizations throughout the FU period of one year.

It is not surprising that creatinine levels were associated with mortality as it has previously been shown that chronic kidney disease is a strong prognostic factor for mortality and cardiovascular disease including stroke, coronary artery disease, heart failure and peripheral arterial disease [23]. Furthermore, it has also been shown that patients with nephrotic syndrome have an increased risk of VTE [24] as well as patients receiving maintenance dialysis and kidney transplant recipients [25,26].

Our findings suggest that the use of DOACs may be safe and non-inferior in efficacy to the standard therapy with LMWH/VKA in the elderly. These findings support those of the subgroup analyses of previous RCTs [1,4,5] as well the results of a previously published systematic review and meta-analysis which showed the same or greater efficacy and safety to DOACs over vitamin K antagonists in elderly patients [16,17,27]. In addition, a recent real-life data analysis of the RIETE registry, in which DOACs were compared to LMWH/VKA in fragile patients (defined as age > 75 or Crcl < 50 mL/min) showed that patients receiving DOACs for long-term therapy had a significantly lower risk for the composite endpoint of recurrent VTE or major bleeding [28]. In another recent analysis of frail patients with VTE rivaroxaban compared to VKA, reduced the composite of recurrent venous thromboembolism or major bleeding [29]. Additionally, a recently pooled analysis of the RE-COVER and RE-COVER II trials found that dabigatran showed better efficacy than warfarin in the elderly [27], in a shorter follow-up period of six months compared to one year in our trial. Several studies conducted in the elderly population with atrial fibrillation have shown similar results: one trial showed lower mortality rates in DOACs patients and similar bleeding events, and in another trial, low dose edoxaban was superior to placebo in preventing stroke and was not associated with higher incidence of major bleeding than placebo. There was no consensus regarding the risk of stroke in these trials [30,31]. In a recent study evaluating very elderly patients (above 85 years) with AF, there were significantly increasing prescription rates of DOACs following the introduction of DOACs [32].

The current study has several limitations. First, the duration of hospitalization was longer by 1.4 days in the VKA/LMWH compared to the DOACs group, a finding that might imply that the difference between the study groups is possibly that patients in the VKA/LMWH group were more severely ill than patients in the DOACs group. Another option is that patients receiving VKA/LMWH were hospitalized for a longer duration for the purpose of reaching therapeutic INR in case of discharging with VKA or to be instructed in injecting in case of discharging with LMWH. However, as mentioned above, in additional analysis of matched cohort, similar results to the original analysis were obtained.

The second limitation stems from the retrospective nature of the study and the potential selection bias associated with it. Information regarding use of the drug by the patient was based on computerized systems and it is difficult to confirm whether the drug was indeed taken with good adherence.

A third limitation is that the DOACs group included a small number of patients;127 patients, which comprised only 21% of the study cohort, compared to the EINSTEIN, RE-COVER and AMPLIFY trials, in which 1731, 1273 and 2691 patients (50% of the study cohort) received rivaroxaban, dabigatran and apixaban, respectively. In our study the small number of patients in the DOAC group may be attributed to the fact that the health regulations in Israel limited use of DOACS in the elderly and for the indication of VTE during part of the study period (treatment with DOACs was included in the Healthcare Basket of Israel for the indication of atrial fibrillation from 2014 and for the indication of VTE from 2018).

A fourth limitation is that we cannot extrapolate the results this study to all types of DOACs because there were no patients treated with edoxaban, since edoxaban was not available for use in Israel.

Another limitation is that there was no information regarding the cause of death in almost 50% of cases.

An additional limitation is that INR levels were not recorded, thus, data regarding time in therapeutic range (TTR) in the VKA group were lacking. The dosages of medications were also not recorded. However, our intent was to examine a real-life situation. Moreover, an Israeli study published in 2015 showed that in patients treated with warfarin for AF, TTR was only 42% of the time [33].

A final limitation is that the study groups were not balanced in comorbidities, and malignancy was more prevalent in the VKA/LMWH group as discussed above. This finding can possibly partly explain the higher rate of mortality in this group. However, a higher percentage of the patients who died from malignancy or malignancy-related complications were treated with DOACs and in a multivariable analysis of the full cohort both malignancy and treatment with VKA/LMWH were independently associated with mortality. As well, in a propensity-matched cohort analysis (in which cancer was one of its parameters), the results were similar.

Our findings have clinical implications. They suggest that in a population of elderly patients, DOACs are equally effective and safe compared to LMWH/VKAs. The disadvantages of daily subcutaneous injections with LMWH and frequent INR monitoring with VKAs are well-known. The variability of INR during warfarin treatment places the patient at a higher risk for bleeding episodes and reduces the effectiveness of the drug. The advantages of direct oral anticoagulants (DOACs) are lower rates of drug interactions, no need of coagulation monitoring and a shorter half-life compared with VKAs. DOACs also have disadvantages such as the dependence on renal elimination, mainly with dabigatran, and the shorter half-life which may become more significant when a tablet is inadvertently omitted.

## 5. Conclusions

In the elderly population, treatment of VTE with DOACs was associated with a lower rate of the composite outcome of all-cause mortality, major bleeding, recurrent VTE and hospitalizations in a follow up of one year. This finding supports the results of previous RCTs. DOACs are safe and effective for elderly patients with VTE, but future RCTs specifically designed for patients >65 years old are recommended.

## Figures and Tables

**Table 1:** Baseline characteristics of patients included in the final analysis.

	VKA Therapy or Enoxaparin (*n* = 476)	DOACs (*n* = 127)	*p*-Value
Age (years), (Mean ± Std)	79.9 ± 8.5	78.6 ± 8.1	0.11
Gender (*n*)	476	127	0.04
Male gender (*n*,%)	213 (44.8)	44 (34.7)	
BMI (Mean ± Std)	27.1 ± 6.4 (*n* = 287)	29.1 ± 11.0 (*n* = 98)	0.08
Co-morbidities			
Myocardial infarction (*n*,%)	86 (18.1)	25 (19.7)	0.70
Congestive heart failure (*n*,%)	50 (10.6)	14 (11.0)	0.87
Peripheral vascular disease (*n*,%)	7 (1.5)	1 (0.8)	1.00
Cerebrovascular disease (*n*,%)	88 (18.5)	27 (21.3)	0.53
Dementia (*n*,%)	38 (8.0)	8 (6.3)	0.71
Chronic pulmonary disease (*n*,%)	62 (13.0)	19 (15.0)	0.56
Connective tissue disease (*n*,%)	20 (4.2)	4 (3.2)	0.80
Peptic ulcer disease (*n*,%)	8 (1.7)	3 (2.4)	0.71
Liver disease (*n*,%)	8 (1.7)	3 (2.4)	0.71
Diabetic mellitus (*n*,%)	117 (24.6)	32 (25.2)	0.91
Renal disease (*n*,%)	22 (4.6)	3 (2.4)	0.32
Malignancy (*n*,%)	96 (20.1)	12 (9.5)	0.004
Acquired Immune Deficiency Syndrome (*n*,%)	0 (0)	0 (0)	
Chronic Medications Prior to the Actual Vte Event *			
DOACs			<0.001
Rivaroxaban (*n*,%)	3 (0.6)	10 (7.9)	
Apixaban (*n*,%)	3 (0.6)	11 (8.7)	
Dabigatran (*n*,%)	4 (0.8)	1 (0.8)	
Antiplatelets			
Aspirin (*n*,%)	181 (38.0)	35 (27.6)	0.03
Clopidogrel (*n*,%)	37 (7.8)	18 (14.2)	0.04
Prasugrel (*n*,%)	1 (0.2)	0 (0)	1.00
Ticagrelor (*n*,%)	1 (0.2)	0 (0)	1.00
Enoxaparin (*n*,%)	56 (11.8)	8 (6.2)	0.05
VKA (*n*,%)	63 (13.2)	9 (7.1)	0.06
**Vital Signs on Admission**			
Systolic BP (Mean ± Std)	134.4 ± 22.7	137.2 ± 25.3	0.23
Diastolic BP (Mean ± Std)	72.5 ± 14.6	73.6 ± 15.2	0.43
Pulse (Mean ± Std)	85.2 ± 17.7	86.5 ± 19.1	0.47
Fever (Mean ± Std)	36.9 ± 0.6	36.9 ± 0.6	0.73
Saturation (Mean ± Std)	94.8 ± 4.1 (*n* = 434)	94.6 ± 5.3 (*n* = 118)	0.71
**Relevant Laboratory Parameters**			
Platelets (Mean ± Std)	245.9 ± 114.9 (*n* = 462)	249.9 ± 98.7 (*n* = 125)	0.70
Leucocytes (Mean ± Std)	12.2± 42.7 (*n* = 462)	11.3 ± 14.2 (*n* = 125)	0.70
Hemoglobin (Mean ± Std)	11.7 ± 1.9 (*n* = 463)	12.4 ± 1.7 (*n* = 125)	<0.001
Creatinine (Mean ± Std)	1.3 ± 1.4 (*n* = 461)	1.1 ± 0.8 (*n* = 125)	0.20
pH (Mean ± Std)	7.4 ± 0.1 (*n* = 203)	7.4 ± 0.1 (*n* = 55)	0.77
D dimer (Mean ± Std)	9807.6 ± 12325.4 (*n* = 52)	11,026.7 ± 10,300.7 (*n* = 11)	0.76
Duration of hospitalization (days), (Median, (Min.Max))	5 (1.50)	4 (2.28)	0.01
Prior VTE (*n*,%)	75 (15.8)	17 (13.4)	0.58
Diagnosis DVT/PE (*n*)	476	127	0.31
DVT (*n*,%)	111 (23.3)	30 (23.6)	
PE (*n*,%)	106 (22.3)	36 (28.4)	
DVT + PE (*n*,%)	259 (54.4)	61 (48.0)	

VKA = Vitamin K antagonist, BMI = body mass index, BP = blood pressure, VTE = venous thromboembolism, DVT = deep vein thrombosis, PE = pulmonary embolism, DOACs = direct oral anticoagulants. * Prior anticoagulation treatment was used as therapy for prior VTE or in other indication such as atrial fibrillation.

**Table 2:** Outcomes.

	VKA/LMWA (*n* = 476)	DOACs (*n* = 127) Rivaroxaban (*n* = 40) Apixaban (*n* = 82) Dabigatran (*n* = 5)
**Composite outcome** (*n* = 427)	355 (74.6%)	72 (56.7%)
**Major bleeding events** (*n* = 35)	32 (6%)	3 (2%)
**Recurrent VTEs** (*n* = 50)	47 (10%)	3 (2%)
**All-cause mortality** (*n* = 316)	300 (63%)	16 (13%)

**Table 3:** Multivariable analysis for the composite outcome of all-cause mortality, major bleeding, recurrent VTE and hospitalizations.

	HR (95%CI)	*p* Value
Treatment group (DOACs vs. VKA/LMWH)	0.64 (0.49–0.83)	0.001
Male	1.05 (0.86–1.28)	0.64
Age	1.02 (1.01–1.04)	0.0001
Baseline Creatinine	1.10 (1.05–1.15)	0.0002
Malignancy	2.26 (1.78–2.87)	<0.0001

HR = Hazard ratio; CI= confidence interval. For continuous variables HR given per 1 unit increment: year (for age), mg/dL (for creatinine).

## Data Availability

The data presented in this study are available on request from the corresponding author. The data are not publicly available due to privacy restriction.

## Supplementary Materials

The following are available online at https://www.mdpi.com/article/10.3390/jcm10235673/s1, Table S1: Causes of death.
