# Supplementary material for: Outcome of Elderly Patients with Venous Thromboembolism Treated with Direct Oral Anticoagulants—A Retrospective Cohort Study
Source: J Clin Med. 2021 Dec 1;10(23):5673. doi: 10.3390/jcm10235673 (PMC8658344; doi:10.3390/jcm10235673)
Supplement: Supplementary file 1 [file jcm-10-05673-s001.zip › jcm-1440848-supplementary.pdf]

Supplemental Table S1. Causes of death.

Cause of Death		Frequency Total Number of Deaths	VKA/DOACs LMWH	Percentage of Cases Out of All Cases of Mortality
**Sepsis**	Pneumonia	41	37<br>4	13.0
	Sepsis with no source	26	26<br>0	8.2
	Soft tissue	5	5<br>0	1.6
	Urosepsis	11	10<br>1	3.5
	Endocarditis	1	1<br>0	0.3
Respiratory failure *		15	14<br>1	4.7
Malignancy or malignancy complication		54	49<br>5	17.1
Renal failure		3	3<br>0	0.9
Heart failure		7	5<br>2	2.2
No information about cause of death **		153	150<br>3	48.4

VKA = vitamin K antagonist, LMWH = low molecular weight heparin, DOACs = direct oral anticoagulants. *Respiratory failure–including pulmonary emboly. **No information about cause of death–the death probably did not take place in a hospital in Israel
